# L-shaped association between oxidative balance score and vision-related functional burden in adults in the United States, NHANES 2005–2008

**DOI:** 10.3389/fnut.2025.1507889

**Published:** 2025-05-02

**Authors:** Juntong Li, Sheng Ye, Xiang Li, Hui Liu, Yue Yang, Xuelei Lu, Heyue Jin, Li Liu

**Affiliations:** ^1^Department of School Health, Nanjing Municipal Center for Disease Control and Prevention Affiliated to Nanjing Medical University, Nanjing, China; ^2^School of Public Health, Nanjing Medical University, Nanjing, China

**Keywords:** oxidative balance score, vision-related functional burden, L-shaped curve, NHANES, adults

## Abstract

**Objective:**

This study aims to investigate the relationship between oxidative balance score (OBS) and vision-related functional burden (VRFB) in US adults.

**Methods:**

The study utilized data from two consecutive cycles of the National Health and Nutrition Examination Survey (NHANES) from 2005 to 2008. A multivariate weighted logistic regression model was employed to explore the relationship between OBS and VRFB in the general population and subgroups, while the interaction effects were tested with a likelihood test. Restricted cubic spline was utilized to assess the nonlinear association of OBS with VRFB. Sensitivity analyses were performed to test the robustness of the results.

**Results:**

A total of 6,682 participants aged 20 years and older were included in the NHANES database. A negative association between OBS and VRFB was observed in the fully adjusted model, with an odds ratio (OR) of 0.968 [95% confidence interval (CI): 0.949–0.987]. Compared with the lowest quartile of OBS, the second (OR: 0.669, 95%CI: 0.486–0.922), the third (OR: 0.589, 95%CI: 0.403–0.859), and highest (OR: 0.554, 95%CI: 0.359–0.855) quartiles of OBS were associated with a reduced risk of VRFB. An L-shaped association was displayed between OBS and VRFB (*P* for nonlinear = 0.016). Notably, when the analysis was stratified by employment status, a significant interaction between OBS and VRFB was observed (*P* for interaction < 0.05). The protective effect of OBS was more pronounced among the unemployed. The results were found to be robust in sensitivity analyses.

**Conclusion:**

This study found an L-shaped relationship between OBS and VRFB among U.S. adults aged 20 years and older. This novel finding suggests that maintaining a favorable oxidative balance through modifiable dietary and lifestyle (such as increased physical activity, smoking cessation, reduced alcohol consumption) may help protect functional vision. We also observed a stronger negative association between OBS and VRFB among the unemployed. This study provides new insights into the prevention of functional vision loss, highlighting the importance of not only dietary and lifestyle factors but also considering different subgroups, such as the unemployed. Further longitudinal studies are required to further validate these observations and elucidate the underlying mechanisms, particularly in elucidating causal relationships among variables, controlling for confounding factors, and examining the development of visual function.

## 1 Introduction

The global prevalence of visual impairment (VI) is a significant public health concern, particularly in light of the aging population. VI refers to the functional limitations of the eye or visual system resulting from various eye diseases, including cataracts, uncorrected refractive errors, glaucoma, age-related macular degeneration (AMD), and diabetic retinopathy (DR) (1, 2). As reported by the World Health Organization (WHO), at least 2.2 billion people were blind or visually impaired worldwide in 2019 (3). It is estimated that by 2050, the number of individuals with moderate to severe VI globally will increase from 216.6 million in 2015 to 587.6 million (4). Visual-related functional burden (VRFB) is used to describe an individual's ability to perform everyday visual tasks in real-world conditions (5). VI has been associated with disabilities in activities of daily living such as reading, household tasks, and driving (6, 7). Previous studies have reported that VI is associated with many chronic diseases, such as chronic kidney disease, type 2 diabetes, sleep disorders, and depressive symptoms (5, 7–9). Seriously, VI can lead to restricted physical activity, significant financial costs, and reduced life expectancy (1, 10).

The human eye, a highly specialized organ of vision, is significantly influenced by oxidants of endogenous and exogenous origin, such as ultraviolet radiation, diseases, aging, environmental pollutants, and other factors (11, 12). Oxidative stress results from an imbalance between the production of reactive oxidative species (ROS) and the body's antioxidant defense system (13). It has the potential to damage tissues, resulting in alterations to tissue structure and function, increased vascular permeability, microvascular abnormalities, and neovascularization. Consequently, these alterations can result in the development of corneal, conjunctival, and optic neuropathy; lens crystallin degeneration; elevated intraocular pressure; and retinal degeneration (14). It represents a significant contributing factor in the pathogenesis of ocular diseases, including AMD, DR, dry eye disease (DED), glaucoma, and cataracts (15–19). Dietary nutrition and lifestyle play a critical role in ocular health. Available evidence suggests that several dietary components, such as vitamins A, B, C, E, carotenoids and magnesium, and lifestyle factors, such as smoking and physical activity, may modulate oxidative stress in AMD and DR (20–22). Collectively, these findings emphasize the critical involvement of oxidative stress in the pathogenesis of ocular diseases. The oxidative balance score (OBS), as a valuable tool for comprehensively measuring oxidative stress-related exposures, incorporates both lifestyle and dietary factors to assess an individual's oxidative balance status (23). The initial OBS was developed by Van Hoydonck et al. and comprises solely three components: two antioxidants (β-carotene and vitamin C) and one pro-oxidant factor (iron) (24). Subsequently, over 20 adaptations of these OBSs have been extensively utilized in epidemiological studies to assess the correlations between oxidative status and the risk of developing chronic diseases (25).

In the field of ophthalmology, previous studies have primarily focused on the mechanisms of occurrence between oxidative stress and specific ocular diseases (11). A recent study has reported a relationship between OBS and DR, with results suggesting that higher OBS is associated with a lower risk of DR (20). However, there is a paucity of epidemiologic evidence on the relationship between OBS and functional vision. In the present study, we collected two consecutive cycles of the National Health and Nutrition Examination Survey (NHANES) data from 2005 to 2008 (2005–2006 and 2007–2008). Our study aims to fill this gap by investigating the relationship between OBS and VRFB and to provide new insights into maintaining eye health in American adults.

## 2 Materials and methods

### 2.1 Study participants

**Figure 1:**
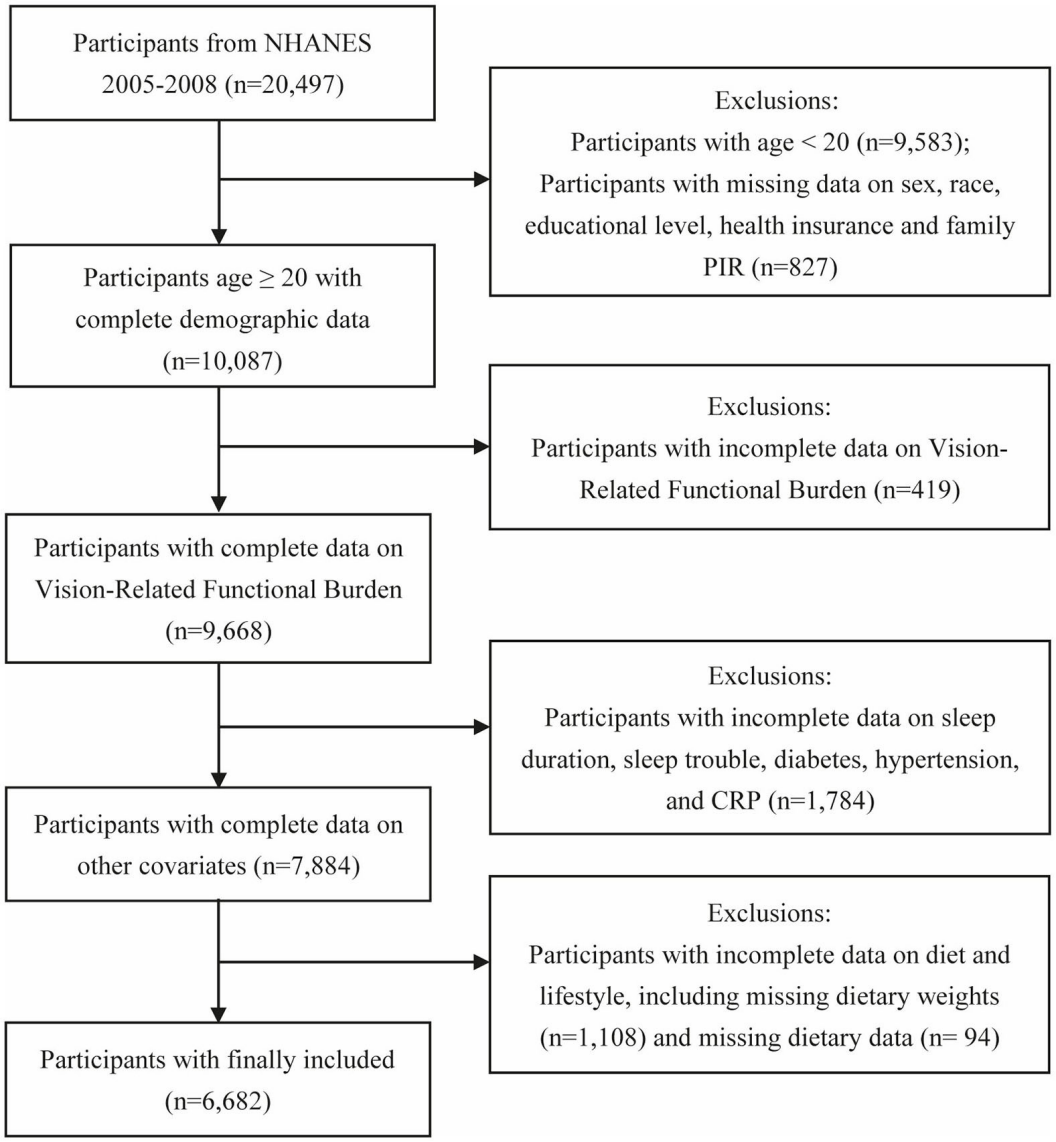
The flow chart of participants' screening.

The NHANES, a periodic health survey program using a stratified, multistage, and probability-cluster design, is designed to assess the health and nutritional status of children and adults in the United States. Figure 1 shows the detailed flow chart of participants screening. A total of 20,497 participants were initially included. The exclusion criteria were set as follows: (1) participants age <20 years (*n* = 9,583), and missing demographic data (*n* = 827); (2) participants with incomplete data on VRFB (*n* = 419); (3) participants with incomplete data on other covariates (*n* = 1,784); (4) participants without relevant information on diet and lifestyle, including missing dietary weights (*n* = 1,108) and missing dietary data (*n* = 94). Following the manual data filtration process, a total of 6,682 participants were ultimately selected for subsequent analyses. The project was approved by the National Center for Health Statistics (NCHS) Research Ethics Review Board, and all participants provided written informed consent. All procedures complied with the principles outlined in the Declaration of Helsinki.

### 2.2 Assessment of OBS

The OBS includes both antioxidants and pro-oxidants, comprising sixteen dietary nutrients and four lifestyle components. The antioxidants are dietary fiber, carotene, vitamin B2, vitamin B6, total folate, vitamin B12, vitamin C, vitamin E, niacin, calcium, magnesium, zinc, copper, selenium, and physical activity (PA). The pro-oxidants include total fat, iron, alcohol consumption, body mass index (BMI), and cotinine. Dietary nutrient intakes and alcohol consumption were evaluated through the mean of 2 days of 24-h dietary recalls. PA was obtained from the physical activity questionnaire, which encompasses daily activities, leisure activities, and sedentary behaviors. Total PA was calculated as the sum of time spent walking and engaging in moderate and vigorous activities per week, classified according to the metabolic equivalent of task levels. Alcohol consumption is the average number of alcoholic beverages of any type consumed per day in the past year. Cotinine, a major metabolite of nicotine, serves as a marker for both active smoking and exposure to environmental tobacco smoke, or “passive smoking.” It is preferred over nicotine for such assessments due to its substantially longer half-life.

The OBS components were evaluated according to the criteria established in previous research. For alcohol consumption, participants were classified into one of three categories: non-drinkers (2 points), light to moderate drinkers (0–15 g/day for women and 0–30 g/day for men, 1 point), and heavy drinkers (≥15 g/day for female and ≥30 g/day for male, 0 points). Other components were scored according to their nature (antioxidant or pro-oxidant) and the participant's sex. Antioxidant components were assigned 0 points for the first tertile, 1 point for the second tertile, and 2 points for the third tertile, whereas pro-oxidant components were scored 2 points for the first tertile, 1 point for the second tertile, and 0 points for the third tertile. The detailed scoring methods for each OBS component are provided in Supplementary Table 1. The overall OBS was calculated by summing the scores of each component, with higher scores indicating a greater predominance of antioxidant exposure.

### 2.3 Assessment of VRFB

Participants aged 20 years and older were asked additional detailed questions about limitations in daily living activities due to their vision. The questions were selected from the National Eye Institute 25-item Visual Functioning Questionnaire, whose reliability and validity have been previously reported (26). VRFB was assessed by six specific questions. The specific questions included: (i) difficulty reading ordinary newsprint, (ii) difficulty with up-close work or chores, (iii) difficulty seeing steps/curbs-dim light, (iv) difficulty noticing objects to the side, (v) difficulty finding objects on a crowded shelf, and (vi) difficulty driving daytime-familiar place. For each category, participants rated their difficulty on a Likert scale: (1) no difficulty, (2) little difficulty, (3) moderate difficulty, (4) extreme difficulty, and (5) unable to do because of eyesight. Participants with VRFB were defined as those who reported moderate difficulty, worse, or unable to do due to eyesight in any of the six questions (27).

### 2.4 Covariates

In our study, demographic variables included sex (male and female), age (categorized into 20–39 years, 40–59 years, and ≥60 years), race (Mexican American, non-Hispanic White, non-Hispanic Black, and other races), educational level (below high school, high school, and college or above), health insurance (no health insurance coverage, public health insurance only, and private health insurance coverage), employment status (unemployment and employment), and family poverty income ratio (family PIR) (regrouped into three levels: ≤ 1.3, 1.3–3.5, and ≥3.5).

Sleep duration was measured by asking, “How much sleep do you usually get at night on weekdays or workdays?” and was categorized into two levels: <7 h and ≥7 h. Sleep trouble and diabetes were based on self-reports of whether participants were told by a doctor or other health professional they have or had sleep trouble, or diabetes, respectively. Hypertension was defined by a systolic BP≥ 140 mmHg and/or diastolic BP≥ 90 mmHg or self-reported hypertension. Energy and omega-3 intake were obtained from a 24-h food recall. Total omega-3 intake was calculated as the sum of alpha-linolenic acid, stearidonic acid, eicosapentaenoic acid, docosapentaenoic acid, and docosahexaenoic acid (28). C-reactive protein (CRP) is a valuable tool for assessing the body's response to chronic inflammatory conditions, such as arthritis, and environmental factors, including tobacco smoke.

### 2.5 Statistical analysis

The sample weights were calculated as 1/2^*^ WTDRD1 (dietary day one sample weight) according to the NHANES analytic and reporting guidelines. Continuous variables were presented as weighted mean (Standard Deviation [SD]), and categorical variables as unweighted frequencies (percentages). Group differences were used in the Kruskal-Wallis test and the Rao-Scott chi-squared test. A weighted logistic regression model was employed to estimate the odds ratio (OR) and 95% confidence intervals (CI) for the association between OBS and VRFB. Initially, OBS was treated as a continuous variable, and subsequently, models were constructed based on OBS quantiles, with participants in the lowest quantile serving as the reference group. We performed linear trend tests by calculating the median value of each quartile of OBS and including these medians as a continuous variable in the model (29). Model 1 was a crude model without adjustment for covariates. Model 2 was adjusted for sex and age. Model 3 was adjusted for sex, age, race, educational level, health insurance, family PIR, employment status, sleep duration, sleep trouble, diabetes, hypertension, energy intake, omega-3, and CRP. To flexibly model and visualize the relation of OBS and VRFB, restricted cubic splines were employed. Additionally, we examined whether these associations varied across different subgroups (age, sex, race, educational level, health insurance, family PIR, employment status, sleep duration, sleep trouble, diabetes, and hypertension) by testing interaction effects using a likelihood ratio test. Moreover, we performed several sensitivity analyses to test the robustness of our results: (i) converting the OBS into tertiles and quintiles; (ii) estimating missing dietary data using the multiple imputation method of chained equations instead of excluding these participants (*n* = 94); and (iii) excluding participants with eye surgery for cataract or myopia (*n* = 1,119).

In this study, all data management and statistical analyses were achieved with R (version 4.2.1, R Foundation for Statistical Computing). The primary R packages, including “survey**,”** “gtsummary**,”** “rms**,”** “jstable**,”** and “ggplot2” were applied. *P* value of <0.05 (two-tailed) was considered statistically significant.

## 3 Results

### 3.1 Characteristics of study participants

This study included 3,289 (47.21%) male and 3,393 (52.79%) female adults aged 20 years and older [with an average age of 46.81(16.51)]. Among these individuals, the majority were non-Hispanic White (74.10%), had a College or above (59.08%), and had private insurance (67.95%). A total of 1,261 individuals (18.87%) were identified as having VRFB. Notably, participants with VRFB tended to be older age, female, absent health insurance, and lower educational level and family PIR. Meanwhile, they exhibited reduced sleep duration, energy intake, omega-3 intake, and OBS. Furthermore, they were more likely to experience sleep trouble, diabetes, and hypertension. Further detailed information can be found in Table 1.

**Table 1:** Characteristics of participants of VRFB.

**Characteristic**	**Overall**	**No**	**Yes**	***P* value**
Participants, *n* (%)	6,682 (100.00)	5,421 (81.13)	1,261 (18.87)	
Age (year), mean (SD)	46.86 (16.51)	45.51 (16.11)	54.64 (16.60)	<0.001
**Sex**, ***n*** **(%)**				0.001
Male	3,289 (47.21)	2,759 (48.49)	530 (39.83)	
Female	3,393 (52.79)	2,662 (51.51)	731 (60.17)	
**Race**, ***n*** **(%)**				0.056
Mexican American	1,119 (7.55)	889 (7.51)	230 (7.80)	
Non-Hispanic White	3,549 (74.10)	2,928 (74.77)	621 (70.23)	
Non-Hispanic Black	1,340 (10.22)	1,073 (9.80)	267 (12.65)	
Other races	674 (8.12)	531 (7.92)	143 (9.32)	
**Educational level**, ***n*** **(%)**				<0.001
Below high school	1,700 (16.49)	1,186 (14.20)	514 (29.70)	
High school	1,604 (24.43)	1,308 (24.09)	296 (26.35)	
College or above	3,378 (59.08)	2,927 (61.70)	451 (43.95)	
**Health insurance**, ***n*** **(%)**				<0.001
No insurance	1,359 (17.40)	1,100 (17.10)	259 (19.14)	
Public insurance	1,334 (14.65)	884 (12.03)	450 (29.76)	
Private insurance	3,989 (67.95)	3,437 (70.87)	552 (51.10)	
**Family PIR**, ***n*** **(%)**				<0.001
≤ 1.3	1,720 (17.32)	1,200 (14.51)	520 (33.48)	
1.3–3.5	2,617 (35.88)	2,114 (35.38)	503 (38.78)	
≥3.5	2,345 (46.80)	2,107 (50.10)	238 (27.74)	
**Employment status**, ***n*** **(%)**				<0.001
Unemployment	1,298 (17.41)	857 (14.39)	441 (34.86)	
Employment	5,384 (82.59)	4,564 (85.61)	820 (65.14)	
**Sleep duration**, ***n*** **(%)**				<0.001
<7	2,571 (35.46)	2,014 (34.20)	557 (42.75)	
≥7	4,111 (64.54)	3,407 (65.80)	704 (57.25)	
Sleep trouble, *n* (%)	1,559 (24.36)	1,098 (21.65)	461 (39.98)	<0.001
Diabetes, *n* (%)	762 (7.80)	502 (6.22)	260 (16.89)	<0.001
Hypertension, *n* (%)	2,880 (38.00)	2,124 (35.02)	756 (55.19)	<0.001
Energy intake (kcal), mean (SD)	2,113.80 (829.81)	2,143.72 (824.73)	1,941.16 (838.29)	<0.001
Omega-3 intake (gm), mean (SD)	1.67 (1.01)	1.69 (1.01)	1.55 (1.02)	<0.001
C-reactive protein (mg/dl), mean (SD)	0.40 (0.76)	0.37 (0.69)	0.57 (1.10)	<0.001
OBS, mean (SD)	20.79 (7.38)	21.15 (7.27)	18.66 (7.63)	<0.001
**OBS (quartiles)**, ***n*** **(%)**				<0.001
Q1 (4–14)	1,862 (24.09)	1,406 (21.97)	456 (36.28)	
Q2 (15–20)	1,573 (22.98)	1,268 (23.15)	305 (22.03)	
Q3 (21–26)	1,715 (26.35)	1,422 (27.04)	293 (22.34)	
Q4 (27–36)	1,532 (26.58)	1,325 (27.84)	207 (19.35)	

### 3.2 Characteristics of participants stratified by quartiles of OBS

Table 2 presents the characteristics of the participants stratified by quartiles of OBS. Participants with higher OBS were observed to be non-Hispanic White, employed, and have a higher educational level and family PIR. Additionally, individuals in the lowest OBS quartile were more likely to report lower sleep duration, lower energy intake, lower omega-3 intake, and higher incidences of sleep trouble, diabetes, and hypertension. They also exhibited elevated levels of CRP.

**Table 2:** Characteristics of participants by quartiles of the OBS.

**Characteristic**	**Overall (6,682)**	**Q1 (1,862)**	**Q2 (1,573)**	**Q3 (1,715)**	**Q4 (1,532)**	***P* value**
Age (year), mean (SD)	46.86 (16.51)	47.55 (17.40)	47.53 (16.75)	46.74 (16.31)	45.77 (15.58)	0.264
**Age**, ***n*** **(%)**						0.077
20–39	2,215 (37.03)	531 (35.77)	509 (36.44)	589 (36.86)	586 (38.86)	
40–59	2,198 (39.24)	583 (37.50)	499 (37.25)	590 (40.79)	526 (40.98)	
≥60	2,269 (23.73)	748 (26.73)	565 (26.30)	536 (22.34)	420 (20.16)	
**Sex**, ***n*** **(%)**						0.053
Male	3,289 (47.21)	920 (45.01)	759 (45.20)	857 (50.06)	753 (48.12)	
Female	3,393 (52.79)	942 (54.99)	814 (54.80)	858 (49.94)	779 (51.88)	
**Race**, ***n*** **(%)**						<0.001
Mexican American	1,119 (7.55)	296 (7.73)	260 (7.62)	301 (8.63)	262 (6.27)	
Non-Hispanic White	3,549 (74.10)	836 (66.84)	817 (74.20)	944 (73.98)	952 (80.71)	
Non-Hispanic Black	1,340 (10.22)	540 (17.53)	338 (10.58)	285 (8.47)	177 (5.03)	
Other races	674 (8.12)	190 (7.89)	158 (7.60)	185 (8.93)	141 (7.99)	
**Educational level**, ***n*** **(%)**						<0.001
Below high school	1,700 (16.49)	659 (25.90)	410 (15.23)	372 (14.91)	259 (10.62)	
High school	1,604 (24.43)	523 (29.45)	387 (27.55)	385 (23.23)	309 (18.37)	
College or above	3,378 (59.08)	680 (44.65)	776 (57.22)	958 (61.86)	964 (71.02)	
**Health insurance**, ***n*** **(%)**						<0.001
No insurance	1,359 (17.40)	441 (23.06)	316 (17.02)	298 (14.75)	304 (15.22)	
Public insurance	1,334 (14.65)	474 (20.25)	306 (13.80)	343 (14.63)	211 (10.32)	
Private insurance	3,989 (67.95)	947 (56.68)	951 (69.18)	1,074 (70.62)	1,017 (74.46)	
**Family PIR**, ***n*** **(%)**						<0.001
≤ 1.3	1,720 (17.32)	640 (26.03)	387 (16.09)	398 (15.72)	295 (12.05)	
1.3-3.5	2,617 (35.88)	772 (39.40)	664 (39.41)	655 (35.58)	526 (29.96)	
≥3.5	2,345 (46.80)	450 (34.57)	522 (44.49)	662 (48.70)	711 (57.99)	
**Employment status**, ***n*** **(%)**						<0.001
Unemployment	1,298 (17.41)	469 (24.74)	282 (15.84)	295 (15.22)	252 (14.30)	
Employment	5,384 (82.59)	1,393 (75.26)	1,291 (84.16)	1,420 (84.78)	1,280 (85.70)	
**Sleep duration**, ***n*** **(%)**						<0.001
<7	2,571 (35.46)	818 (42.17)	626 (37.52)	630 (34.01)	497 (29.05)	
≥7	4,111 (64.54)	1,044 (57.83)	947 (62.48)	1,085 (65.99)	1,035 (70.95)	
Sleep trouble, *n* (%)	1,559 (24.36)	460 (26.42)	372 (24.67)	380 (22.60)	347 (23.97)	0.345
Diabetes, *n* (%)	762 (7.80)	267 (9.53)	199 (9.22)	192 (8.24)	104 (4.55)	<0.001
Hypertension, *n* (%)	2,880 (38.00)	943 (43.64)	712 (40.35)	677 (35.35)	548 (33.49)	0.001
Energy intake (kcal), mean (SD)	2,113.80 (829.81)	1,506.04 (514.22)	1,892.57 (567.70)	2,239.32 (686.44)	2,731.33 (912.04)	<0.001
Omega-3 intake (gm), mean (SD)	1.67 (1.01)	1.11 (0.59)	1.52 (0.86)	1.76 (0.93)	2.23 (1.19)	<0.001
C-reactive protein, mean (SD) (mg/dl)	0.40 (0.76)	0.50 (0.88)	0.43 (0.89)	0.35 (0.61)	0.32 (0.65)	<0.001

### 3.3 Association between OBS and VRFB

**Figure 2:**
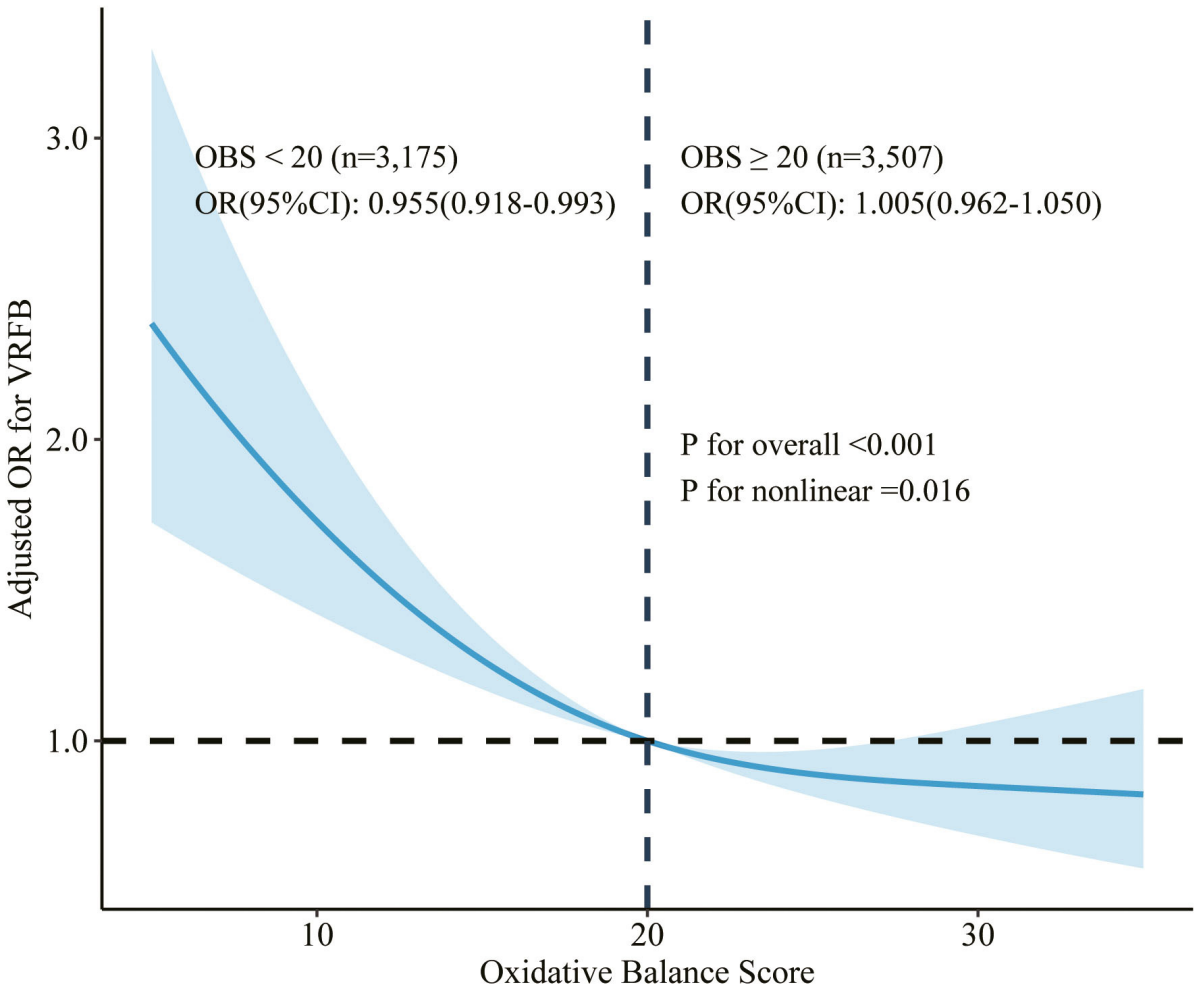
Restricted cubic spline curve for the association between OBS and VRFB, adjusting for age, sex, race, education level, health inusurance, family PIR, employment status, sleep duration, sleep trouble, diabetes, hypertension, energy intake, omega-3, and CRP.

Three sampling-weighted multivariate logistic regression models were constructed to examine the relationship between OBS and VRFB in this study (Table 3). A negative association between OBS and VRFB in the fully adjusted model, with an OR of 0.968 [95% CI: 0.949–0.987], indicating that the risk of VRFB was reduced with each unit increase in OBS. In addition, in comparison to the lowest quartile of OBS, the second (OR: 0.669, 95%CI: 0.486–0.922), the third (OR: 0.589, 95%CI: 0.403–0.859), and highest (OR: 0.554, 95%CI: 0.359–0.855) quartiles of OBS were associated with a reduced risk of VRFB, with all *P* values for trend being <0.05. As shown in Figure 2, an L-shaped association was displayed between OBS and VRFB (*P* for nonlinear = 0.016). Specifically, a negative correlation was observed between OBS and VRFB when OBS was <20 (OR: 0.955; 95% CI: 0.918–0.993). However, this trend was not statistically significant when OBS exceeded 20 (OR: 1.055; 95% CI: 0.962–1.050).

**Table 3:** Association of OBS with VRFB.

**OBS**	**Model 1**	**Model 2**	**Model 3**
Continue	0.955 (0.942–0.968)	0.957 (0.943–0.972)	0.968 (0.949–0.987)
Q1	Ref	Ref	Ref
Q2	0.576 (0.449–0.739)	0.579 (0.441–0.759)	0.669 (0.486–0.922)
Q3	0.500 (0.377–0.664)	0.514 (0.387–0.683)	0.589 (0.403–0.859)
Q4	0.421 (0.319-0.556)	0.449 (0.328–0.614)	0.554 (0.359–0.855)
*P* for trend	<0.0001	<0.0001	0.0076

Ref: the least quartile (Q1) as the reference group.

Model 1 was a crude model without adjusting covariates.

Model 2 was adjusted for age and sex.

Model 3 was adjusted for age, sex, race, educational level, health insurance, family PIR, employment status, sleep duration, sleep trouble, diabetes, hypertension, energy intake, omega-3, and CRP.

### 3.4 Subgroup and sensitivity analyses

**Figure 3:**
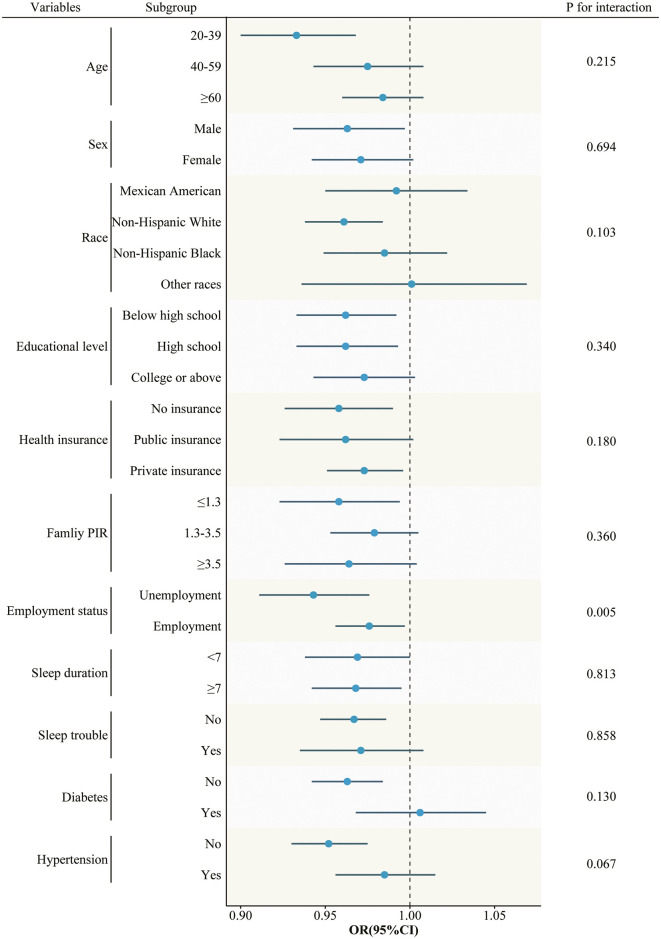
Subgroup analyses of the association between OBS and VRFB.

The results were consistent across a wide range of subgroups stratified by age, sex, race, educational level, health insurance, family PIR, sleep duration, sleep trouble, diabetes, and hypertension (Figure 3). Notably, when the analysis was stratified by employment status, a significant interaction between OBS and VRFB was observed (*P* for interaction <0.05). The protective effect of OBS was more pronounced among the unemployed. However, when OBS was treated as a quartile, no significant interaction was observed (Supplementary Figure 1). The sensitivity analyses based on tertiles and quintiles of OBS yielded consistent results (Supplementary Table 2). Furthermore, the utilization of the multiple imputation method of chained equations for the estimation of missing dietary data yielded robust results (Supplementary Table 3). Similar results were observed when we excluded participants with eye surgery for cataracts or myopia (Supplementary Table 4).

## 4 Discussion

In this national, multiracial, cross-sectional study of 6,682 participants in the United States, a negative correlation was found between OBS and VRFB among adults aged 20 years and older. This is consistent with previous studies in DR populations (20). Notably, an L-shaped association was observed between OBS and VRFB. Subgroup analyses demonstrated that this phenomenon was particularly prevalent among the employed population. Furthermore, the sensitivity analyses provided further evidence to support the robustness of the results.

In this study, the OBS incorporates both pro-oxidant and antioxidant factors, as well as lifestyle and dietary variables, to assess an individual's oxidative balance status. Pro-oxidant factors can induce oxidative stress through the generation of ROS or by reducing antioxidant defense activity. In contrast, antioxidant factors have the potential to shift the balance toward a less pro-oxidant state (30). Notable antioxidant factors include dietary antioxidants, maintaining a healthy weight, refraining from smoking, engaging in regular physical activity, and abstaining from alcohol consumption (31).

The VRFB showed differences for OBS components (Supplementary Table 5). In addition, we further explored the association between each component of OBS, dietary OBS, lifestyle OBS, and VRFB (Supplementary Tables 6–8, and Supplementary Figures 2, 3). A cross-sectional study across multiple regions has demonstrated that nutrients are of paramount importance in maintaining vision health (21). Essential vitamins including A, C, E, and beta-carotene, in conjunction with enzyme systems localized within various cellular compartments—including mitochondria, peroxisomes, and the cytosol—work synergistically with key minerals such as selenium, copper, and zinc to support the eye's defense mechanisms against oxidative stress and promote overall ocular health (12, 32). A diet that is rich in antioxidants is of great importance to support these innate defense systems. Vitamin C, as a potent antioxidant, plays a crucial role in ocular physiology and the prevention of eye diseases (33). It protects ocular structures, such as the lens and cornea, from oxidative damage, thereby enhancing antioxidant defenses. A meta-analysis revealed a significant association between lower levels of vitamin C and DR (34). Vitamin E has been demonstrated to combat free radicals, prevent lipid oxidation, and maintain cell membrane integrity, while also serving to shield retinal ganglion cells from oxidative stress (35, 36). It also reduces the risk of cataracts by protecting the lens of the eye from oxidative damage (37). Additionally, Vitamin E contributes to the antioxidant defenses of the ocular surface, potentially benefiting patients with DED (38). The AREDS also indicated that 5 years after the end of the clinical trial, the beneficial effects of the antioxidants C, E, β-carotene, and/or zinc on the development of AMD persisted (38). The vitamin B complex plays a critical role in maintaining overall eye health and reducing the risk of diseases such as cataracts and glaucoma (39). In this study, the dietary OBS components include vitamins B2, B3, B6, and B12, which possess antioxidant properties and promote neuronal survival and regeneration (40). The AREDS found a negative correlation between increased intake of B2 and B12 and the incidence of cataracts (41). The available evidence suggests that antioxidant vitamin and mineral supplements may have the potential to slow the progression of AMD (42).

Beyond dietary factors, it is important to consider the impact of lifestyle on functional vision. Obesity is a risk factor for most potentially blinding eye diseases, such as glaucoma, cataracts, AMD, and DR (43, 44). Inflammation and oxidative stress play significant role in the relationship between obesity and ocular diseases (45). Sedentary behavior increases the risk of dry eye symptoms, particularly in individuals with low levels of physical activity (46). A higher level of physical activity is associated with a reduced incidence of cataracts due to a reduction in oxidative damage (43). Animal research indicated that aerobic exercise may safeguard photoreceptor cells and retinal pigment epithelium from harm and prevent thinning of the retinal and photoreceptor layers in a mouse model of retinal degeneration (47). Smoking and alcohol consumption lower antioxidant levels in the body, leading to additional oxidative damage (48). One study found that rats exposed to cigarette smoke exhibited damage to the cornea and lacrimal glands, which may be related to ROS-induced DNA oxidation (49). Several studies have revealed that alcohol consumption is associated with primary open-angle glaucoma, DR, and AMD (50, 51).

In the subgroup analyses, we identified a significant interaction between employment status and the relationship between OBS and VRFB. Notably, the protective effect of OBS was more pronounced among the unemployed, suggesting a potential modifying influence of OBS within this group. This interaction has important implications for understanding how different subgroups may benefit differently from interventions aimed at preventing functional vision loss. Similar studies have indicated that individuals with lower socioeconomic status tend to have poorer dietary and lifestyle habits, as well as a higher prevalence of eye diseases (52, 53). Therefore, it is crucial to strengthen health education and promote lifestyle improvements, particularly for those with lower socioeconomic status.

The present study has several strengths that enhance the validity and reliability of the findings. First, the utilization of data from the nationally representative survey NHANES permits the results to be generalized to a more expansive US population. The weighted analysis guarantees that the resulting estimates are representative of the entire population and takes into account the complex survey design. Furthermore, the OBS incorporates both antioxidants and pro-oxidants, integrating dietary and lifestyle variables for a more comprehensive assessment of individual antioxidant status. Secondly, the restricted cubic spline analysis offered valuable insights into the non-linear association between the OBS and VRFB. A further strength of this study is the sensitivity analysis, which assesses the stability of the results.

However, some limitations to this study should be recognized. First, this cross-sectional study provides valuable insights into the association between OBS and VRFB; however, it faces limitations in causal inference. For instance, the presence of chronic vision problems may result in a decline in quality of life, which, in turn, could contribute to the development of oxidative stress. Consequently, the necessity for additional longitudinal studies is evident in order to establish the causal relationship between these two variables. Second, VRFB was assessed by answering six questions on functional visual impairment, which could have introduced subjective bias in assessment. Future studies should use objective measurement methods to assess visual function, as this would enhance the reliability of the research. Third, the 24-h dietary recall method used to collect dietary data may introduce recall bias, as it relies on the participants' ability to accurately remember and report their food intake. Future studies could improve the accuracy of dietary recall data by utilizing multiple 24-h recall periods and incorporating other validation methods. Fourth, potential confounding factors such as screen use duration, other disease histories, and medication histories were not fully considered, and overlooking these factors could introduce bias in interpreting the relationship. It is recommended that more prospective studies explore these factors in more detail to improve the accuracy and generalizability of the results. Fifth, a further limitation of OBS is that it is unable to measure endogenous antioxidant or pro-oxidant function. Moreover, our analyses are based on the assumption that the impact of pro- and antioxidant exposures is equally weighted, which may not accurately reflect the actual contribution of each exposure. Finally, the data utilized in this study were derived from the NHANES between 2005 to 2008. The release of an updated version of the NHANES questionnaire on functional vision is eagerly anticipated. In future studies, it is necessary to adopt a longitudinal follow-up research design to explore the association between oxidative balance status and functional vision. The core assessment framework includes two major dimensions: (1) oxidative balance status indicators, which are measured using biomarkers such as plasma total antioxidant capacity and glutathione peroxidase activity; and (2) visual function assessment indicators, encompassing high-contrast visual acuity and contrast sensitivity function.

## 5 Conclusion

In conclusion, we found an L-shaped relationship between OBS and VRFB among U.S. adults aged 20 years and older, where higher antioxidant exposure, as measured by the OBS, was associated with a reduced risk of VRFB. This novel finding suggests that maintaining a favorable oxidative balance through modifiable dietary and lifestyle (such as increased physical activity, smoking cessation, reduced alcohol consumption) may help protect functional vision. We also observed a stronger negative association between OBS and VRFB among the unemployed. This study provides new insights into the prevention of functional vision loss, highlighting the importance of not only dietary and lifestyle factors but also considering different subgroups, such as the unemployed. Further longitudinal studies are required to further validate these observations and elucidate the underlying mechanisms, particularly in elucidating causal relationships among variables, controlling for confounding factors, and examining the development of visual function.

## Data Availability

The datasets presented in this study can be found in online repositories. The names of the repository/repositories and accession number(s) can be found at: https://www.cdc.gov/nchs/nhanes/.
