# Supplementary material for: L-shaped association between oxidative balance score and vision-related functional burden in adults in the United States, NHANES 2005–2008
Source: Front Nutr. 2025 May 2;12:1507889. doi: 10.3389/fnut.2025.1507889 (PMC12082837; doi:10.3389/fnut.2025.1507889)
Supplement: Supplementary file 1 [file Table_1.docx]

Supplementary Material

# Supplementary Figures and Tables

This supplementary material provides more details on the statistical analysis results conducted for interested readers.

## Supplementary Figures


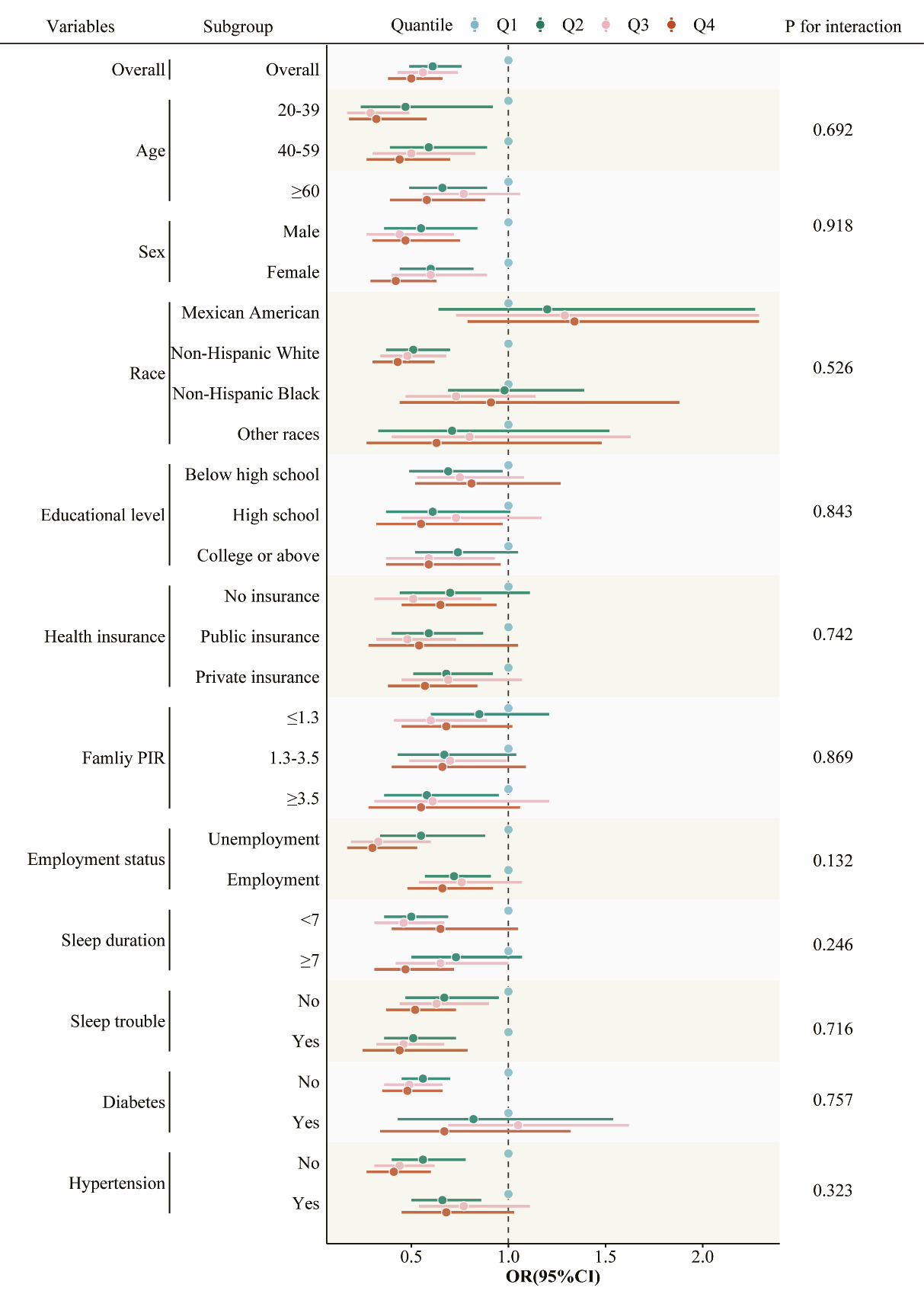


Supplementary Figure 1

Subgroup analysis of the association between OBS quantiles (the lowest quantile as the reference group) and VRFB, adjusting for age, sex, race, educational level, health insurance, family PIR, employment status, sleep duration, sleep trouble, diabetes, hypertension, energy intake, omega-3, and CRP.


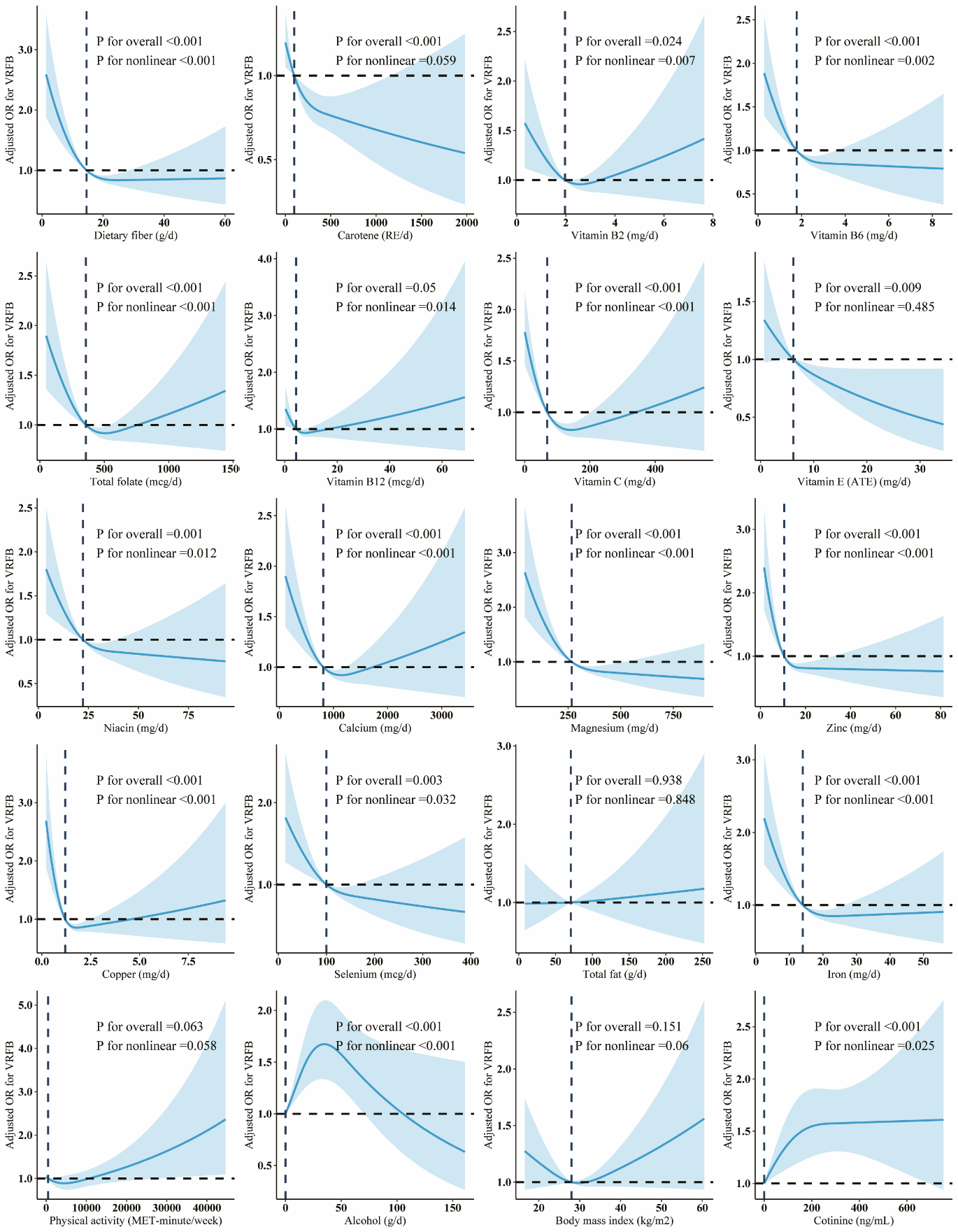


Supplementary Figure 2

Restricted cubic spline curve for the association between every component of OBS and VRFB, adjusting for age, sex, race, educational level, health insurance, family PIR, employment status, sleep duration, sleep trouble, diabetes, hypertension, energy intake, omega-3, and CRP.


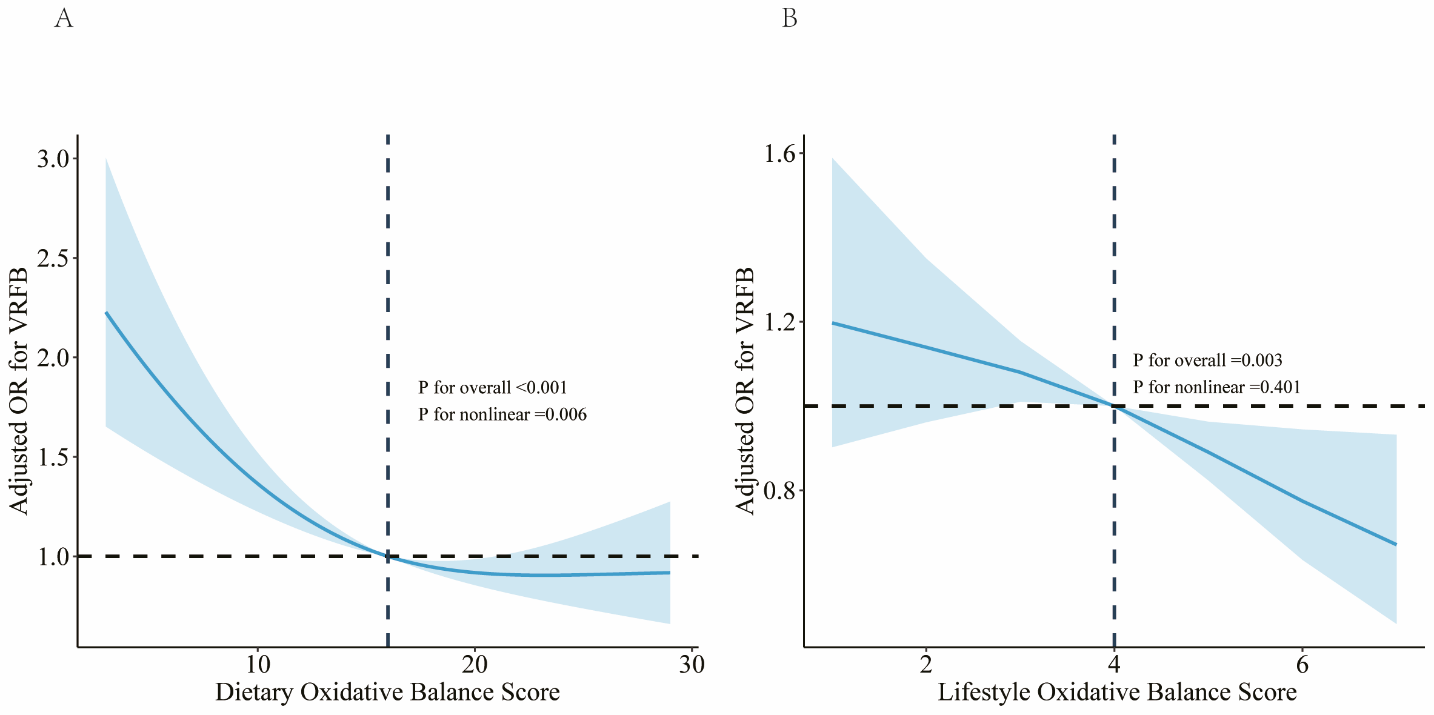


Supplementary Figure 3

Restricted cubic spline curve for the association between dietary OBS, lifestyle OBS, and VRFB. (A) dietary OBS with VEFB; (B) lifestyle OBS with VEFB. Models were adjusted for age, sex, race, educational level, health insurance, family PIR, employment status, sleep duration, sleep trouble, diabetes, hypertension, energy intake, omega-3, and CRP.

## Supplementary Tables

Supplementary Table 1 The oxidative balance score (OBS) assignment scheme.

| OBS components | Property | Male | | | Female | | |
| --- | --- | --- | --- | --- | --- | --- | --- |
|  |  | 0 | 1 | 2 | 0 | 1 | 2 |
| Dietary fiber (g/d) | A | <12.90 | 12.90-19.80 | >19.80 | <10.95 | 10.95-16.35 | >16.35 |
| Carotene (RE/d) | A | <53.54 | 53.54-177.03 | >177.03 | <54.19 | 54.19-191.73 | >191.73 |
| Vitamin B2 (mg/d) | A | <1.84 | 1.84-2.68 | >2.68 | <1.45 | 1.45-2.09 | >2.09 |
| Vitamin B6 (mg/d) | A | <1.65 | 1.65-2.51 | >2.51 | <1.28 | 1.28-1.88 | >1.88 |
| Total folate (mcg/d) | A | <328.00 | 328.00-489.50 | >489.50 | <265.00 | 265.00-388.17 | >388.17 |
| Vitamin B12 (mcg/d) | A | <3.87 | 3.87-6.58 | >6.58 | <2.75 | 2.75-4.88 | >4.88 |
| Vitamin C (mg/d) | A | <46.20 | 46.20-108.87 | >108.87 | <44.35 | 44.35-94.25 | >94.25 |
| Vitamin E (ATE) (mg/d) | A | <5.53 | 5.53-8.52 | >8.52 | <4.50 | 4.50-6.94 | >6.94 |
| Niacin (mg/d) | A | <21.66 | 21.66-31.42 | >31.42 | <16.16 | 16.16-22.96 | >22.96 |
| Calcium (mg/d) | A | <719.00 | 719.00-1077.17 | >1077.17 | <609.00 | 609.00-920.00 | >920.00 |
| Magnesium (mg/d) | A | <254.83 | 254.83-350.00 | >350.00 | <201.83 | 201.83-282.67 | >282.67 |
| Zinc (mg/d) | A | <10.13 | 10.13-14.95 | >14.95 | <7.45 | 7.45-11.04 | >11.04 |
| Copper (mg/d) | A | <1.12 | 1.12-1.56 | >1.56 | <0.91 | 0.91-1.27 | >1.27 |
| Selenium (mcg/d) | A | <98.03 | 98.03-138.77 | >138.77 | <72.67 | 72.67-102.22 | >102.22 |
| Total fat (g/d) | P | >101.00 | 67.89-101.00 | <67.89 | >74.42 | 50.69-74.42 | <50.69 |
| Iron (mg/d) | P | >19.39 | 13.09-19.39 | <13.09 | >14.80 | 10.25-14.80 | <10.25 |
| PA (MET-minute/week) | A | <240.00 | 240.00-1890.00 | >1890.00 | <80.00 | 80.00-945.00 | >945.00 |
| Alcohol consumption (g/d) | P | >=30 | 0-30 | None | >=15 | 0-15 | None |
| Body mass index (kg/m2) | P | >30.05 | 26.01-30.05 | <26.01 | >31.11 | 25.38-31.11 | <25.38 |
| Cotinine (ng/mL) | P | >2.23 | 0.04-2.23 | <0.04 | >0.16 | 0.02-0.16 | <0.02 |

OBS: oxidative balance score;

A: antioxidant;

P: prooxidant;

RE: retinol equivalent;

ATE: alpha-tocopherol equivalent;

MET: metabolic equivalent.

Supplementary Table 2: Conversion of the OBS into tertiles and quintiles;

Supplementary Table 3: Estimation of missing dietary data using the multiple imputation method of chained equations (MICE) in place of exclusion (n=94);

Supplementary Table 4: Exclusion of participants with eye surgery for cataract or myopia (n=1,119).

Supplementary Table 2 Association between OBS and VRFB.

| OBS | Model 1 | Model 2 | Model 3 |
| --- | --- | --- | --- |
| Continue | 0.955(0.942-0.968) | 0.957(0.943-0.972) | 0.968(0.949-0.987) |
| Tertile 1 | Ref | Ref | Ref |
| Tertile 2 | 0.550(0.407-0.743) | 0.561(0.408-0.771) | 0.607(0.414-0.888) |
| Tertile 3 | 0.467(0.372-0.585) | 0.490(0.383-0.627) | 0.580(0.421-0.800) |
| *P* for trend | <0.0001 | <0.0001 | 0.0028 |
|  |  |  |  |
| Quintitle 1 | Ref | Ref | Ref |
| Quintitle 2 | 0.757(0.604-0.948) | 0.767(0.600-0.982) | 0.914(0.665-1.255) |
| Quintitle 3 | 0.422(0.305-0.585) | 0.429(0.305-0.603) | 0.499(0.324-0.769) |
| Quintitle 4 | 0.491(0.375-0.643) | 0.512(0.387-0.676) | 0.659(0.445-0.975) |
| Quintitle 5 | 0.442(0.334-0.586) | 0.471(0.343-0.646) | 0.623(0.395-0.984) |
| *P* for trend | <0.0001 | <0.0001 | 0.0096 |

Supplementary Table 3 Association between OBS and VRFB.

| OBS | Model 1 | Model 2 | Model 3 |
| --- | --- | --- | --- |
| Continue | 0.953(0.940-0.966) | 0.956(0.942-0.971) | 0.967(0.949-0.987) |
| Quartile 1 | Ref | Ref | Ref |
| Quartile 2 | 0.580(0.458-0.735) | 0.591(0.451-0.775) | 0.668(0.471-0.947) |
| Quartile 3 | 0.491(0.365-0.660) | 0.507(0.376-0.683) | 0.594(0.394-0.893) |
| Quartile 4 | 0.416(0.317-0.547) | 0.454(0.334-0.618) | 0.557(0.351-0.883) |
| *P* for trend | <0.0001 | <0.0001 | 0.0122 |

Supplementary Table 4 Association between OBS and VRFB.

| OBS | Model 1 | Model 2 | Model 3 |
| --- | --- | --- | --- |
| Continue | 0.959(0.943-0.975) | 0.961(0.944-0.977) | 0.969(0.948-0.991) |
| Quartile 1 | Ref | Ref | Ref |
| Quartile 2 | 0.576(0.430-0.771) | 0.579(0.420-0.798) | 0.685(0.467-1.005) |
| Quartile 3 | 0.507(0.369-0.696) | 0.512(0.374-0.702) | 0.579(0.385-0.871) |
| Quartile 4 | 0.463(0.339-0.634) | 0.490(0.349-0.686) | 0.597(0.370-0.962) |
| *P* for trend | <0.0001 | <0.0001 | 0.0165 |

Ref: the least tertile or quartile as the reference group.

Model 1 was a crude model with unadjusted covariates.

Model 2 was adjusted for age and sex.

Model 3 was adjusted for age, sex, race, educational level, health insurance, family PIR, employment status, sleep duration, sleep trouble, diabetes, hypertension, energy intake, omega-3, and CRP.

Supplementary Table 5 Comparison of OBS Components by VRFB.

| Characteristic | Total | No | Yes | *P* value |
| --- | --- | --- | --- | --- |
| Dietary fiber (g/d) | 16.23 (8.22) | 16.51 (8.22) | 14.62 (8.04) | <0.001 |
| Carotene (RE/d) | 197.97 (250.17) | 202.31 (254.31) | 172.92 (223.25) | 0.002 |
| Riboflavin (mg/d) | 2.25 (1.07) | 2.27 (1.05) | 2.11 (1.13) | <0.001 |
| Vitamin B6 (mg/d) | 2.05 (1.10) | 2.09 (1.10) | 1.82 (1.04) | <0.001 |
| Total folate (mcg/d) | 414.02 (208.46) | 420.18 (207.57) | 378.52 (210.15) | <0.001 |
| Vitamin B12 (mcg/d) | 5.62 (5.28) | 5.67 (5.08) | 5.32 (6.31) | <0.001 |
| Vitamin C (mg/d) | 87.32 (76.72) | 89.18 (76.99) | 76.60 (74.26) | <0.001 |
| Vitamin E (ATE) (mg/d) | 7.58 (4.65) | 7.74 (4.70) | 6.68 (4.20) | <0.001 |
| Niacin (mg/d) | 25.50 (12.27) | 26.01 (12.29) | 22.51 (11.68) | <0.001 |
| Calcium (mg/d) | 941.06 (489.64) | 955.73 (485.80) | 856.39 (503.16) | <0.001 |
| Magnesium (mg/d) | 297.17 (127.61) | 302.25 (127.15) | 267.84 (126.35) | <0.001 |
| Zinc (mg/d) | 12.45 (8.50) | 12.68 (8.64) | 11.16 (7.53) | <0.001 |
| Copper (mg/d) | 1.37 (0.79) | 1.39 (0.73) | 1.27 (1.06) | <0.001 |
| Selenium (mcg/d) | 111.50 (50.60) | 113.55 (50.64) | 99.70 (48.77) | <0.001 |
| Total fat (g/d) | 80.67 (38.10) | 81.75 (37.98) | 74.41 (38.16) | <0.001 |
| Iron (mg/d) | 15.93 (7.86) | 16.19 (7.86) | 14.38 (7.68) | <0.001 |
| PA (MET-minute/week) | 2,448.23 (4,938.96) | 2,498.28 (4,955.36) | 2,159.45 (4,835.19) | <0.001 |
| Alcohol consumption (g/d) | 8.88 (20.58) | 9.03 (20.76) | 8.00 (19.50) | 0.015 |
| Body mass index (kg/m2) | 28.65 (6.62) | 28.47 (6.40) | 29.68 (7.65) | 0.006 |
| Cotinine (ng/mL) | 63.42 (131.87) | 58.77 (127.56) | 90.25 (151.72) | <0.001 |

Supplementary Table 5 provides a detailed comparison of OBS components based on VRFB. The values are presented as mean (sd). This comparison helps elucidate the relationship between dietary and lifestyle factors influencing oxidative stress and VRFB.

Supplementary Table 6 Association between every component of OBS and VRFB.

| Variable | OR(95%CI) | *P* value |
| --- | --- | --- |
| Dietary fiber (g/d) | 0.974(0.957-0.991) | **0.0060** |
| Carotene (RE/d) | 0.999(0.999-1.000) | **0.0124** |
| Riboflavin (mg/d) | 0.985(0.885-1.095) | 0.7580 |
| Vitamin B6 (mg/d) | 0.863(0.778-0.957) | **0.0090** |
| Total folate (mcg/d) | 1.000(0.999-1.000) | 0.2746 |
| Vitamin B12 (mcg/d) | 1.002(0.982-1.022) | 0.8282 |
| Vitamin C (mg/d) | 0.998(0.996-1.000) | **0.0212** |
| Vitamin E (mg/d) | 0.965(0.937-0.993) | **0.0178** |
| Niacin (mg/d) | 0.987(0.977-0.997) | **0.0157** |
| Calcium (mg/d) | 1.000(0.999-1.000) | 0.3641 |
| Magnesium (mg/d) | 0.998(0.997-1.000) | **0.0112** |
| Zinc (mg/d) | 0.984(0.955-1.013) | 0.2470 |
| Copper (mg/d) | 0.952(0.755-1.201) | 0.6545 |
| Selenium (mcg/d) | 0.997(0.993-1.000) | 0.0686 |
| Total fat (g/d) | 1.001(0.994-1.008) | 0.7975 |
| Iron (mg/d) | 0.981(0.965-0.998) | **0.0323** |
| PA (MET-minute/week) | 1.000(1.000-1.000) | 0.5380 |
| Alcohol consumption (g/d) | 1.003(0.998-1.009) | 0.1892 |
| Body mass index (kg/m2) | 1.004(0.989-1.020) | 0.5653 |
| Cotinine (ng/mL) | 1.001(1.000-1.002) | **0.0074** |

A multivariate weighted logistic regression model was employed to explore the relationship between every component of OBS and VRFB, adjusting for age, sex, race, educational level, health insurance, family PIR, employment status, sleep duration, sleep trouble, diabetes, hypertension, energy intake, omega-3, and CRP.

Supplementary Table 7 Association between dietary OBS and VRFB.

| Dietary OBS | Model 1 | Model 2 | Model 3 |
| --- | --- | --- | --- |
| Continue | 0.959(0.945-0.973) | 0.964(0.948-0.980) | 0.964(0.945-0.984) |
| Quartile 1 | Ref | Ref | Ref |
| Quartile 2 | 0.638(0.546-0.745) | 0.633(0.536-0.747) | 0.675(0.530-0.858) |
| Quartile 3 | 0.503(0.382-0.663) | 0.532(0.399-0.708) | 0.570(0.392-0.829) |
| Quartile 4 | 0.497(0.398-0.621) | 0.546(0.421-0.709) | 0.578(0.405-0.826) |
| *P* for trend | <0.0001 | 0.0001 | 0.0035 |

Supplementary Table 8 Association between lifestyle OBS and VRFB.

| Lifestyle OBS | Model 1 | Model 2 | Model 3 |
| --- | --- | --- | --- |
| Continue | 0.790(0.739-0.845) | 0.792(0.743-0.843) | 0.911(0.848-0.979) |
| Quartile 1 | Ref | Ref | Ref |
| Quartile 2 | 0.725(0.551-0.955) | 0.729(0.543-0.978) | 0.932(0.689-1.260) |
| Quartile 3 | 0.575(0.439-0.753) | 0.573(0.425-0.773) | 0.814(0.610-1.087) |
| Quartile 4 | 0.370(0.265-0.518) | 0.370(0.263-0.519) | 0.632(0.444-0.898) |
| *P* for trend | <0.0001 | <0.0001 | 0.0062 |

Ref: the least tertile or quartile as the reference group.

Model 1 was a crude model with unadjusted covariates.

Model 2 was adjusted for age and sex.

Model 3 was adjusted for age, sex, race, educational level, health insurance, family PIR, employment status, sleep duration, sleep trouble, diabetes, hypertension, energy intake, omega-3, and CRP.
